# Androgen Deprivation Therapy and Cardiovascular Risk

**DOI:** 10.5812/numonthly.7597

**Published:** 2012-12-15

**Authors:** Panagiotis Mourmouris, Eleni Efstathiou, Athanasios Papatsoris

**Affiliations:** 1 Department of Urology, School of Medicine, University of Athens, Sismanoglio General Hospital, Athens, Greece; 2 Department of Clinical Therapeutics / Oncology, School of Medicine, University of Athens, Alexandra Hospital, Athens, Greece

**Keywords:** Androgen Antagonists, Androgens, Prostatic Neoplasms, Cardiovascular system

**Figure 1:**
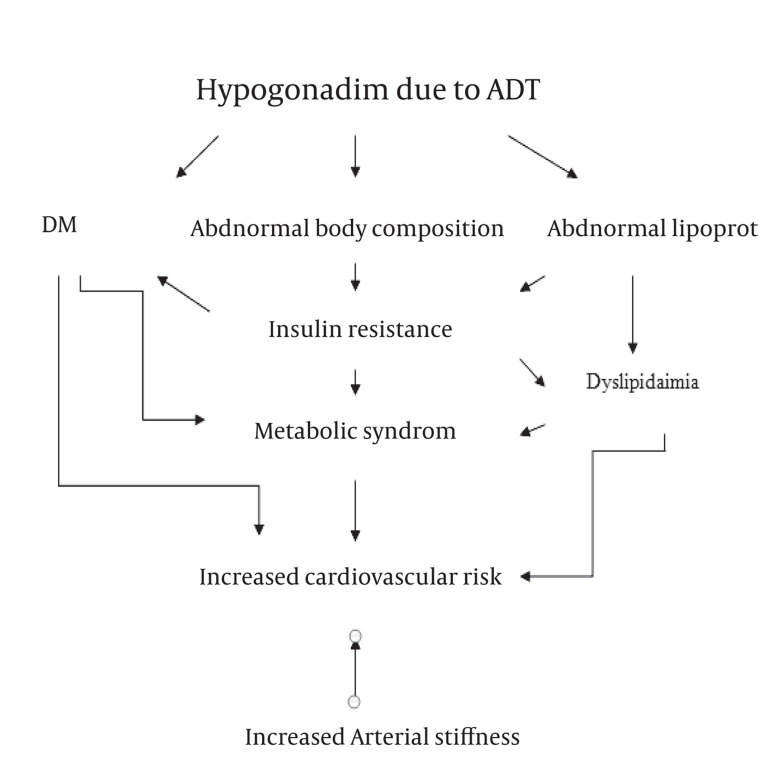
Mechanisms of Increased Cardiovascular Risk During ADT

Androgen deprivation therapy (ADT) is an established therapy for metastatic prostate cancer (PC) and some cases of locally advanced and/or localized PC (1). However, concerns have been raised about the cardiovascular side effects of ADT and their impact on the survival of elderly patients with PC (1). Several studies have demonstrated an increased incidence of coronary heart disease, heart failure and acute myocardial infarction in patients on ADT. For instance, in a study on 1015 patients that received ADT (mean duration: 4.1 months), the use of ADT statistically significantly increased the risk of death from cardiovascular causes (HR = 2.6, P = 0.002) (2). In another study on 22816 patients with PC, multivariate analysis revealed that ADT significantly increased cardiovascular morbidity (3). Regardless of the studies that indicate the correlation of ADT with increased cardiovascular risk, surprisingly little is known on the potential mechanisms. ADT increases insulin concentration despite unchanged plasma glucose, which is suggestive of insulin resistance (4). Peripheral resistance to insulin can induce or precipitate type 2 diabetes mellitus (DM) and metabolic syndrome (4). Furthermore, ADT changes the body mass composition as it leads to muscular atrophy and an increase in subcutaneous fat, a situation characterized as “sarcopenic obesity” (5). A study in patients on ADT (mean duration: 3 months) has shown a 4.3% increase in fat mass and a 1.4% decrease in lean body mass (6). Moreover, studies have demonstrated that ADT is associated with dyslipidemias, lower levels of high density lipoprotein and higher levels of triglycerides, total cholesterol and low density lipoprotein concentrations (7). Furthermore, Chen et al. (8) revealed that long-term ADT (mean duration: 2.5 years) significantly decreased the levels of apolipoproteins I and II. Lastly, Nishiyama et al. (9) demonstrated that after 6 months of ADT, body weight, levels of fasting blood sugar, serum total cholesterol, blood urea nitrogen, compensated calcium, inorganic phosphorus, bone-specific alkaline phosphatase, and compensated urinary deoxypyridinoline increased significantly Arterial stiffness increase is another potential mechanism explaining the increased cardiovascular risk in patients on ADT. In a relevant study, arterial stiffness was assessed with pulse-wave analysis (10). After 3 months of ADT, the augmentation index increased from 24% to 29% (P = 0.003), while the timing of wave reflection was reduced from 137 to 129 msec (P = 0.003). Fat mass increased from 20.2 to 21.9 kg (P = 0.008), whereas lean body mass decreased from 63.2 to 61.5 kg (P = 0.016). In a subgroup of patients whose treatment was discontinued after 3 months, the augmentation index decreased from 31% at month to 29% at month 6, in contrast to patients receiving continuous ADT, where the augmentation index remained elevated at month 6 (P = 0.043). These results have been confirmed by other relevant studies (11). In the following diagram (Figure 1) we propose potential mechanisms contributing to the increased cardiovascular risk in patients on ADT. Relevant studies are ongoing and their results are warranted.
